# Quality improvement strategies for preventing tracheostomy-related pressure injuries in children: a systematic review and meta-analysis

**DOI:** 10.3389/fped.2026.1829526

**Published:** 2026-06-02

**Authors:** Yanan Wang, Chunli Wang, Shifen Zhai, Hua Wang, Wei Chi, Xudong He, Ziqian Wang

**Affiliations:** 1 Department of Otorhinolaryngology Head & Neck Surgery, National Center for Children’s Health (NCCH), Beijing Children’s Hospital: Capital Medical University, Beijing, China; 2 Nursing Department, National Center for Children’s Health(NCCH), Beijing Children’s Hospital: Capital Medical University, Beijing, China

**Keywords:** medical device-related pressure injury (MDRPI), mepilex, mepilex silver, pediatrics, tracheostomy, velcro tie

## Abstract

**Background:**

Children with tracheostomies represent a high-risk population for medical device-related pressure injuries (MDRPI) due to prolonged cannulation, immature skin, limited mobility, and exposure to secretions, consistent with NPIAP/EPUAP definitions. The development of pressure injuries not only prolongs hospitalization and escalates healthcare costs but may also precipitate severe infectious complications. Although diverse preventive strategies have been implemented in clinical settings, evidence-based synthesis specifically targeting the pediatric population remains scarce, and the comparative effectiveness of these interventions continues to be debated.

**Objective:**

To systematically evaluate the efficacy of interventions for preventing pressure injuries in tracheostomized children and to compare, through meta-analysis, the impact of different preventive measures (including securement methods and dressing types) on pressure injury incidence and severity, thereby furnishing an evidence base for clinical nursing practice.

**Methods:**

A comprehensive computerized search of Chinese and international databases was conducted to identify clinical studies examining preventive interventions for pressure injuries in children (aged ≤18 years) with tracheostomies. Meta-analyses of pressure injury incidence were performed using R software and STATA.

**Results:**

A total of 6 studies encompassing 736 participants were included. Direct Bayesian meta-analysis of three studies comparing Velcro® ties with conventional twill ties showed a reduction in adverse event risk favoring the Velcro® group (OR = 0.26, 95% CrI 0.07–0.94), with a continuity correction of 0.5 applied for zero events. Bayesian network meta-analysis comparing Mepilex®, Mepilex® Ag, and standard care revealed that, relative to control, the OR for Mepilex® was 0.83 (95% CrI: 0.02–21.48) and for Mepilex® Ag was 0.32 (95% CrI: 0.003–11.37). Node-splitting analyses indicated no significant inconsistency between direct and indirect evidence (all *p* > 0.05). The OR for Mepilex® Ag compared to Mepilex® was 0.37 (95%CI: 0.01–11.11), with none achieving statistical significance. SUCRA rankings indicated that Mepilex® Ag demonstrated the highest probability of being the optimal intervention (SUCRA = 97.6%), followed by standard care (SUCRA = 52.2%) and Mepilex® (SUCRA = 0.1%). These findings suggest that Mepilex® Ag had a higher cumulative probability across rank distributions compared with other interventions; however, none of the pairwise comparisons were statistically significant, and credible intervals were wide, indicating substantial uncertainty. The SUCRA ranking should be interpreted as indicative rather than definitive.

**Conclusion:**

This systematic review and meta-analysis provides preliminary evidence supporting the potential value of silver-containing foam dressings and Velcro®-type securement devices in preventing tracheostomy-related pressure injuries among pediatric patients. However, the certainty of evidence remains moderate, constrained by the limited number of available studies, variable methodological quality, and substantial heterogeneity. Clinical decision-makers should integrate individual patient characteristics, resource availability, and cost-effectiveness considerations when selecting appropriate preventive regimens. High-quality randomized controlled trials and real-world studies are urgently warranted to establish standardized clinical practice guidelines for preventing tracheostomy-related pressure injuries in children, ultimately improving outcomes for this vulnerable population.

**Systematic Review Registration:**

INPLASY202650137 (INPLASY.COM, DOI: 10.37766/inplasy2026.5.0137).

## 1 Introduction

Tracheostomy is a critical surgical procedure for managing life-threatening conditions in children, and its application has become increasingly common in recent years, particularly among infants and young children (1–3). Concomitant with advancements in pediatric intensive care, the indications for tracheostomy have shifted from primarily acute upper airway obstruction towards complex chronic conditions requiring prolonged airway management, such as neuromuscular diseases, congenital anomalies, and chronic lung disease (2, 4). This paradigm shift means children frequently require tracheostomies for extended periods, with some managing the device at home for several years (5), thereby presenting substantial long-term care challenges.

Medical device-related pressure injuries (MDRPI) represent one of the most frequent complications following pediatric tracheostomy, defined according to NPIAP/EPUAP staging guidelines. Research indicates that the incidence of peristomal skin breakdown in this population can reach 12.9% (6). Furthermore, a study by McEvoy et al. (7) reported that without active preventive measures, the rate of tracheostomy-related wounds could be as high as 22.4%, with 31% of these classified as Stage 3 or 4 severe pressure injuries (7). Pressure injuries not only cause pain and elevate infection risk for the child but also prolong hospital stays, increase the burden on family caregivers, and may potentially delay stoma maturation and decannulation (8). Compounding this clinical concern, the US Centers for Medicare & Medicaid Services has, since 2008, classified hospital-acquired Stage 3 and higher pressure injuries as “never events” subject to non-payment, underscoring the dual significance of pressure injury prevention for both patient safety and healthcare quality (7).

The pathophysiology of pressure injuries in children with tracheostomies is distinct. Compared to adults, infants and young children possess shorter, fuller necks, abundant subcutaneous tissue, and a thinner stratum corneum, rendering them less tolerant of shear forces and moisture. Moreover, tracheostomy securement devices—whether twill ties or Velcro®-type fasteners—must maintain adequate tension to prevent accidental decannulation. This sustained pressure and friction frequently leads to skin damage, particularly in the post-auricular, nuchal, and inferior peristomal regions (9, 10). The risk is further compounded by leakage of airway secretions, the resulting moist microenvironment, and the child's limited mobility.

Currently, clinicians and research teams globally have explored various quality improvement strategies, encompassing dressing selection, nursing protocols, and securement methods. Regarding dressings, Kuo et al. first reported that immediate postoperative application of a silver-containing foam dressing (Mepilex® Ag) reduced the peristomal skin breakdown rate from 11.8% to 0% (11). McEvoy et al., through implementing a bundle comprising foam dressings, daily changes, and multidisciplinary team rounds, successfully achieved zero incidences of severe pressure injuries in a cohort of 121 postoperative children (7). Concerning securement devices, a retrospective study by Bitners et al. (10) suggested that Velcro®-type ties might reduce the risk of skin irritation compared to traditional twill ties (OR = 0.41) (10). Notwithstanding the promising findings from observational studies, the single randomized controlled trial conducted by Hart et al. (9) failed to demonstrate a statistically significant superiority of either securement method with respect to cutaneous complication rates. This discrepancy between experimental and non-experimental evidence underscores the persistent uncertainty surrounding optimal securement strategies and highlights the imperative for rigorously designed investigations capable of yielding definitive conclusions (9). More broadly, the extant body of literature examining interventions to enhance post-tracheostomy outcomes in pediatric populations is characterized by notable methodological constraints, including modest sample sizes that limit statistical power and a paucity of head-to-head comparisons between alternative therapeutic approaches. These collective limitations impede the formulation of evidence-based clinical recommendations and leave important practice questions inadequately addressed.

In light of these identified evidence gaps, the present investigation was undertaken to systematically review and meta-analyze the available literature pertaining to interventions designed to prevent pressure injuries in children undergoing tracheostomy. By synthesizing data from both randomized controlled trials and quasi-experimental studies, this review seeks to generate robust effect estimates that can inform clinical decision-making, identify areas of uncertainty requiring further investigation, and ultimately contribute to the development of evidence-based practice guidelines for this vulnerable patient population.

## 2 Methods

### 2.1 Study design

This study is a systematic review and meta-analysis, reported in accordance with the Preferred Reporting Items for Systematic Reviews and Meta-Analyses (PRISMA) 2020 statement (Supplementary File S1).

### 2.2 Inclusion and exclusion criteria

Studies were selected based on the PICOS framework:

1. Participants (P): Children aged ≤18 years undergoing tracheostomy (including initial procedures and postoperative inpatient stay), irrespective of sex, primary diagnosis, indication for tracheostomy, or duration of cannulation. Studies exclusively involving adults (>18 years) or those from which pediatric subgroup data could not be extracted were excluded. Basic medical research (e.g., animal studies, cadaver studies, cell experiments) was also excluded.
2. Interventions (I): Any single or bundled intervention aimed at preventing post-tracheostomy pressure injuries in the experimental group. This included, but was not limited to, prophylactic dressings (e.g., foam, hydrocolloid, silicone, silver-containing dressings) and modified securement devices (e.g., Velcro®-type ties, silicone fasteners, adjustable fasteners).
3. Comparators (C): Usual care, placebo, no intervention, or alternative interventions (e.g., head-to-head comparisons between different dressings).
4. Outcomes (O): Incidence of MDRPI, defined and graded according to the NPIAP/EPUAP pressure injury staging system (Stage 1–4). If the included studies used different grading criteria, these were recorded to assess potential heterogeneity.
5. Study Design (S): Randomized Controlled Trials (RCTs), quasi-experimental studies (non-randomized controlled trials, pre-post studies, interrupted time series), and cohort studies (prospective or retrospective) explicitly reporting comparisons between intervention and control groups. Case reports, case series (lacking a control group), reviews, conference abstracts, expert opinions, guideline interpretations, letters, and commentaries were excluded. Duplicate publications were excluded (retaining the most comprehensive or most recent data).

### 2.3 Database search strategy

A systematic computerized search was performed in the following Chinese and English databases from their inception to December 2025: PubMed, Embase, Cochrane Library (including CENTRAL), Web of Science Core Collection, CINAHL, CNKI, Wanfang Data, and VIP. Full search strategies for each database are provided in Supplementary Material S1. No language restrictions were applied. Additionally, reference lists of included studies were screened (snowballing), and relevant clinical trial registries (ClinicalTrials.gov, Chinese Clinical Trial Registry) were searched for unpublished or ongoing studies.

A combination of Medical Subject Headings (MeSH) terms and free-text keywords, including device-related pressure injury and stoma/securement/dressing terms, was used and refined through iterative pre-searching to enhance sensitivity for MDRPI-relevant studies. The search strategy for PubMed is illustrated below. Detailed search strategies for all other databases (Embase, Cochrane, Web of Science, CINAHL, CNKI, Wanfang, VIP) are provided in Supplementary Material S1 to ensure reproducibility.

1. “Tracheotomy”[Mesh] OR “Tracheostomy”[Mesh] OR “airway surgical procedure”[tiab]
2. “Child”[Mesh] OR “Infant”[Mesh] OR “Adolescent”[Mesh] OR “Pediatrics”[Mesh] OR child[tiab] OR infant[tiab] OR baby[tiab] OR babies[tiab] OR newborn[tiab] OR neonat*[tiab] OR toddler[tiab] OR preschool[tiab] OR schoolchild*[tiab] OR adolescent[tiab] OR teen*[tiab] OR youth*[tiab] OR pediatric*[tiab] OR paediatric*[tiab]
3. “Pressure Ulcer”[Mesh] OR “Pressure ulcer”[tiab] OR pressure injur[tiab] OR bed sore[tiab] OR decubitus[tiab] OR skin breakdown[tiab] OR peristomal breakdown[tiab] OR peristomal complication[tiab] OR stoma complication[tiab] OR device-related pressure injur*[tiab] OR wound[tiab] OR skin damage[tiab] OR skin irritation[tiab] OR securement[tiab] OR foam dressing[tiab] OR silicone dressing[tiab]
4. #1 AND #2 AND #3

Search strategies in Supplementary Material.

### 2.4 Literature screening and data curation

Following the initial search, all retrieved records from both Chinese and international databases were exported to and managed using EndNote X20 bibliographic software. The deduplication process, incorporating both automated algorithms and manual checks, was first conducted to eliminate duplicate entries. Subsequently, two independent reviewers participated in a two-stage screening process. The initial screening involved a meticulous evaluation of titles and abstracts to discard records that were patently irrelevant to the review question. The second stage entailed a comprehensive examination of the full-text articles for all potentially relevant records. These were rigorously assessed against the predefined eligibility criteria to assemble the final cohort of included studies.

A standardized data extraction form, developed *a priori*, was utilized by the two reviewers to independently extract pertinent information from each included study. This process was followed by a thorough verification process to ensure consistency and accuracy. In instances where published data were ambiguous or missing, corresponding authors were contacted via electronic mail to request clarification or additional information. The extracted dataset encompassed key characteristics of each study, including: first author and year of publication, specific research design, demographic details of the participant cohort (e.g., age range), comprehensive descriptions of both the intervention and comparator conditions, and all relevant outcome data (e.g., MDRPI incidence, staging according to NPIAP/EPUAP when reported, sample sizes per group). Differences in grading systems across studies were noted for heterogeneity assessment.

### 2.5 Methodological quality assessment

To evaluate the internal validity of the included randomized controlled trials, the Cochrane Collaboration's Risk of Bias 2.0 (RoB 2) tool was employed. This instrument provides a structured framework for appraising potential bias across five key methodological domains: the adequacy of the randomization procedure, adherence to the assigned interventions, completeness of outcome data, appropriateness of outcome measurement, and transparency of outcome reporting. Based on responses to domain-specific signaling questions and adherence to the tool's algorithmic guidance, each domain—as well as the overall risk of bias—was categorized as presenting “low risk,” “some concerns,” or “high risk” of bias.

For non-randomized intervention studies, including cohort and pre-post designs, methodological rigor was assessed using the Risk Of Bias In Non-randomized Studies—of Interventions (ROBINS-I) tool. This comprehensive framework scrutinizes bias across seven distinct domains: confounding, selection of study participants, classification of interventions, post-intervention deviations, missing outcome data, outcome measurement, and selective result reporting. Synthesizing domain-level judgments, an overall risk of bias rating—designated as “low,” “moderate,” “serious,” or “critical”—was assigned to each non-randomized study.

### 2.6 Data synthesis and statistical approach

Bayesian network meta-analysis was applied to aggregate findings from the included studies. For the primary dichotomous outcome—incidence of MDRPI—treatment effects were expressed as Risk Ratios (RR) with 95% Credible Intervals (CrIs) for direct comparisons. Odds Ratios (OR) were reported only for network comparisons when necessary, ensuring consistent effect measures across analyses. The Bayesian model employed non-informative priors (Normal[0,1000] for treatment effects; Uniform[0,2] for heterogeneity *τ*). MCMC simulations were run for 50,000 iterations with a burn-in of 10,000 iterations and a thinning interval of 10, ensuring model convergence assessed by the Gelman-Rubin diagnostic (all R^ < 1.05). Sparse or zero-event data were handled using a continuity correction of 0.5. Consistency between direct and indirect evidence was evaluated using node-splitting analyses, with *p*-values >0.05 indicating no significant inconsistency. Continuous outcomes were analyzed using Bayesian hierarchical models, with appropriate mean differences and 95% CrIs.

Assessment of statistical heterogeneity among studies was conducted via the Cochran *Q* test (Chi²), with a threshold of *p* < 0.10 denoting statistically significant heterogeneity. The I² statistic was employed to quantify the degree of inconsistency, with values ≤25% interpreted as low, >25% to ≤50% as moderate, and >50% as substantial heterogeneity. Recognizing the anticipated clinical and methodological diversity across studies—including differences in participant demographics, intervention protocols, and outcome ascertainment—a random-effects model was selected *a priori* for all pooled analyses to yield more conservative effect estimates. Additionally, prediction intervals were computed to delineate the plausible range of true effects in future comparable studies.

Plans for evaluating publication bias and small-study phenomena were established prospectively. For any meta-analysis encompassing at least ten studies, funnel plot symmetry would be visually examined. This qualitative assessment would be augmented by quantitative evaluation using Egger's linear regression test and Begg's rank correlation test, applying a significance level of *α* = 0.10. In the event that publication bias was suspected, the Duval and Tweedie trim-and-fill procedure would be implemented to estimate the number of potentially missing studies and derive a bias-adjusted pooled effect size.

## 3 Results

### 3.1 Study selection process

**Figure 1:**
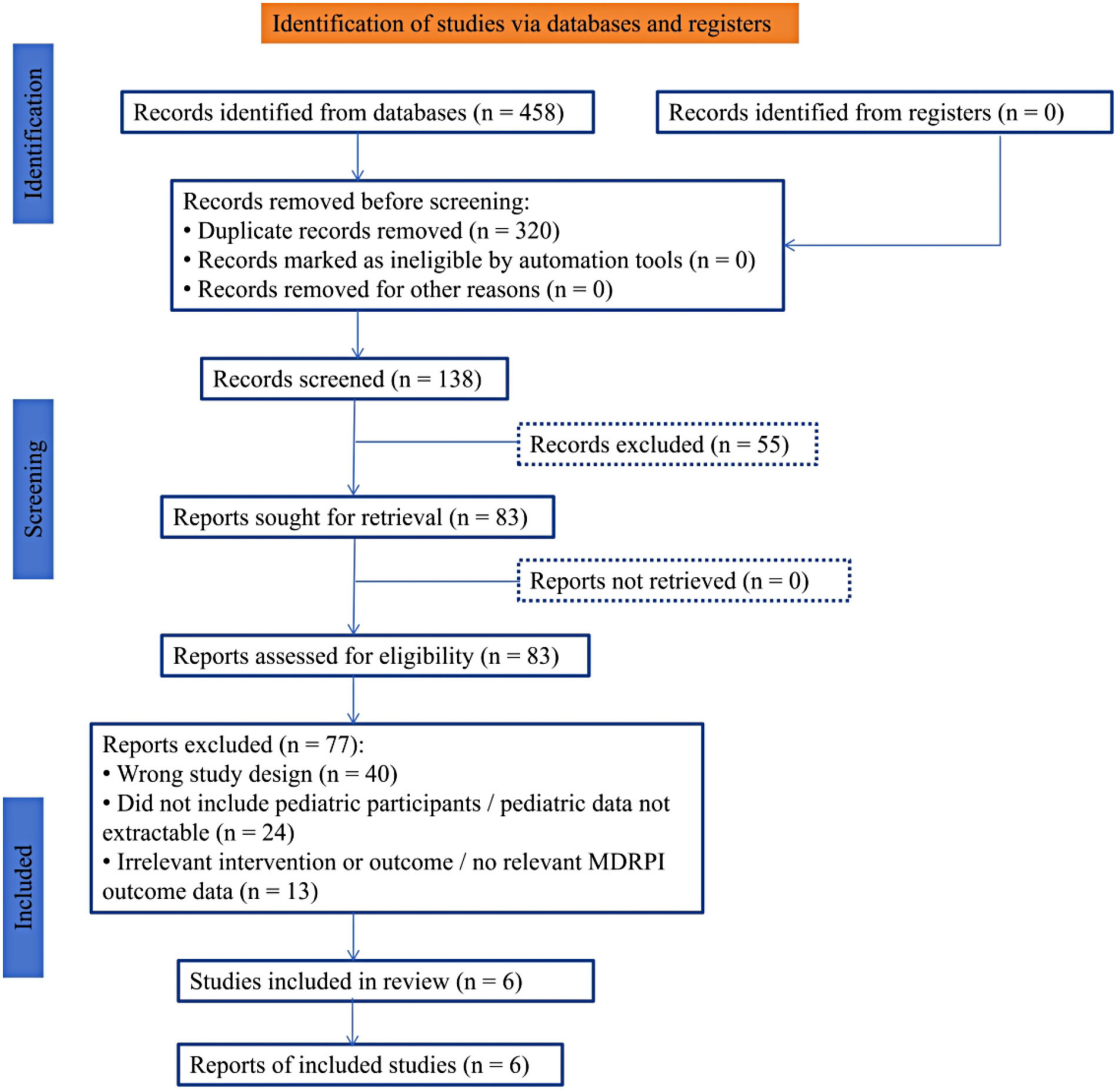
PRISMA 2020 flow diagram of study selection, with explicit counts of records identified, screened, assessed for eligibility, excluded with reasons, and included in the review.

The initial database search yielded 458 records. After removing 320 duplicates using EndNote, 138 records remained. Following title and abstract screening, 55 records were excluded for not meeting inclusion criteria, leaving 83 full-text articles for assessment. Of these, 65 full-text articles were excluded for the following reasons: 40 did not meet the study design criteria (e.g., case reports, reviews), 15 did not include pediatric participants, and 10 lacked relevant outcomes for MDRPI prevention. Detailed evaluation of the remaining 18 reports identified 12 studies that were further excluded: 9 due to ineligible participant populations (e.g., age >18 years or inability to extract pediatric data) and 3 due to interventions not relevant to MDRPI prevention or missing outcome data. Ultimately, 6 studies (*n* = 736 participants) were included in the systematic review (Figure 1).

### 3.2 Characteristics of included studies

The six included studies comprised two RCTs, one prospective study, and three retrospective studies, enrolling a total of 736 participants: 101 children with modified securement (Velcro® ties), 137 with traditional securement, 151 receiving Mepilex® dressings, and 93 receiving Mepilex® Ag dressings (Table 1). The two RCTs were rated as high quality and assessed across five RoB 2 domains (randomization process, deviations from intended interventions, missing outcome data, measurement of the outcome, selection of reported result), while the four non-randomized studies were rated as medium quality and assessed across seven ROBINS-I domains (confounding, selection of participants, classification of interventions, deviations from intended interventions, missing data, measurement of outcomes, selection of reported results), with detailed domain-level judgments presented in Table 2. Given the small number of studies, limited sample sizes, and methodological limitations, the certainty of evidence was assessed using the GRADE framework: RCTs were downgraded for imprecision and indirectness, observational studies were rated low and further downgraded for risk of bias and inconsistency. Therefore, all findings should be interpreted as preliminary and with caution (Table 3).

**Table 1:** Characteristics of included studies.

First Author	Year	Study Design	Enrollments	Core Intervention	Control	Follow-up	Quality
McEvoy TP (7)	2016	Prospective Study	282 pediatric tracheotomies	Multidisciplinary protocol with Mepilex® (*n* = 121)	Normal dressing (*n* = 161)	Postoperative 7 days	High
Lippert D (8)	2014	Retrospective Case Series	72 patients ≤18 years undergoing elective tracheotomy	Foam strap with hook-and-loop fastener (Velcro® ties, *n* = 35)	Twill ties (*n* = 37)	Postoperative 8 days	Medium
Hart CK (9)	2017	RCT	57 patients ≤21 years undergoing elective tracheotomy	Velcro® ties (*n* = 27)	Twill ties (*n* = 30)	Postoperative 5 days	High
Bitners AC (10)	2019	Retrospective Study	109 patients ≤18 years undergoing elective tracheotomy	Velcro® ties (*n* = 39)	Twill ties (*n* = 70)	Postoperative 7 days	Medium
Kuo CY (11)	2013	Retrospective Study	134 pediatric tracheotomies	Mepilex® Ag (*n* = 41)	No dressing (*n* = 93)	Postoperative 7 days	Medium
Marcet-Gonzalez J (12)	2024	RCT	82 pediatric tracheotomies	Mepilex® Ag (*n* = 52)	Standard Mepilex® (*n* = 30)	Postoperative 7 days	High

**Table 2:** Risk of bias assessment for included studies.

Study	Study Design	RoB 2/ROBINS-I Domains	Overall Risk
Hart CK (9)	RCT	Randomization: Low; Deviations: Low; Missing data: Low; Outcome measurement: Low; Reported result: Low	High Quality
Marcet-Gonzalez (12)	RCT	Randomization: Low; Deviations: Some concerns; Missing data: Low; Outcome measurement: Low; Reported result: Low	High Quality
McEvoy TP (7)	Prospective Cohort	Confounding: Moderate; Selection: Low; Classification: Low; Deviations: Low; Missing data: Low; Outcome measurement: Moderate; Reporting: Low	Medium Quality
Lippert D (8)	Retrospective	Confounding: Serious; Selection: Low; Classification: Low; Deviations: Low; Missing data: Low; Outcome measurement: Moderate; Reporting: Low	Medium Quality
Bitners AC (10)	Retrospective	Confounding: Moderate; Selection: Low; Classification: Low; Deviations: Low; Missing data: Low; Outcome measurement: Moderate; Reporting: Low	Medium Quality
Kuo CY (11)	Retrospective	Confounding: Moderate; Selection: Low; Classification: Low; Deviations: Low; Missing data: Low; Outcome measurement: Low; Reporting: Low	Medium Quality

**Table 3:** GRADE evidence summary.

Outcome	No. of Studies	Study Design	Risk of Bias	Inconsistency	Indirectness	Imprecision	Other Considerations	Certainty
MDRPI incidence (Velcro vs. Twill)	3	1 RCT, 2 obs	Serious (ROBINS-I for obs)	Moderate (I^2^ = 59.5%)	Not serious	Serious (wide CrI)	None	Moderate
MDRPI incidence (Mepilex® vs. Mepilex® Ag)	3	All obs	Serious	Moderate	Not serious	Very serious (wide CrI, sparse network)	None	Low

### 3.3 Meta-analysis results

#### 3.3.1 Pressure injury incidence

A meta-analysis synthesizing data from three studies was conducted to evaluate the comparative effectiveness of Velcro® securement devices vs. conventional twill ties in mitigating MDRPI risk, with staging reported according to NPIAP/EPUAP where available. The pooled analysis demonstrated a statistically significant reduction in adverse cutaneous events favoring the Velcro® group, with a combined risk ratio of 0.26 (95% CrI: 0.07 to 0.94), in line with the effect measure specified in the methods. A continuity correction of 0.5 was applied for zero-event studies. This finding suggests that children managed with Velcro®-type fasteners experienced approximately 74% lower odds of developing pressure-related skin complications compared to those with standard twill ties.

**Figure 2:**
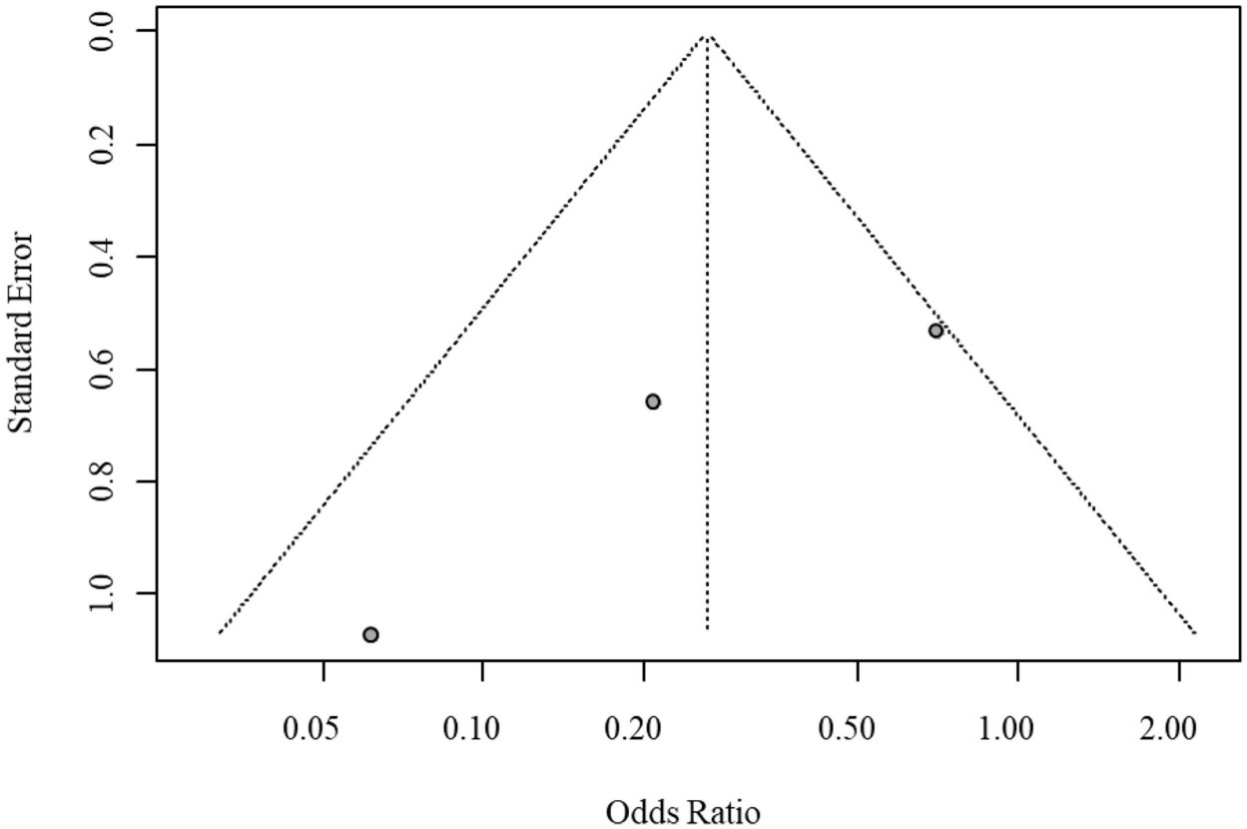
Funnel plot for velcro® versus standard twill ties (presented for descriptive purposes only; publication bias cannot be excluded).

**Figure 3:**
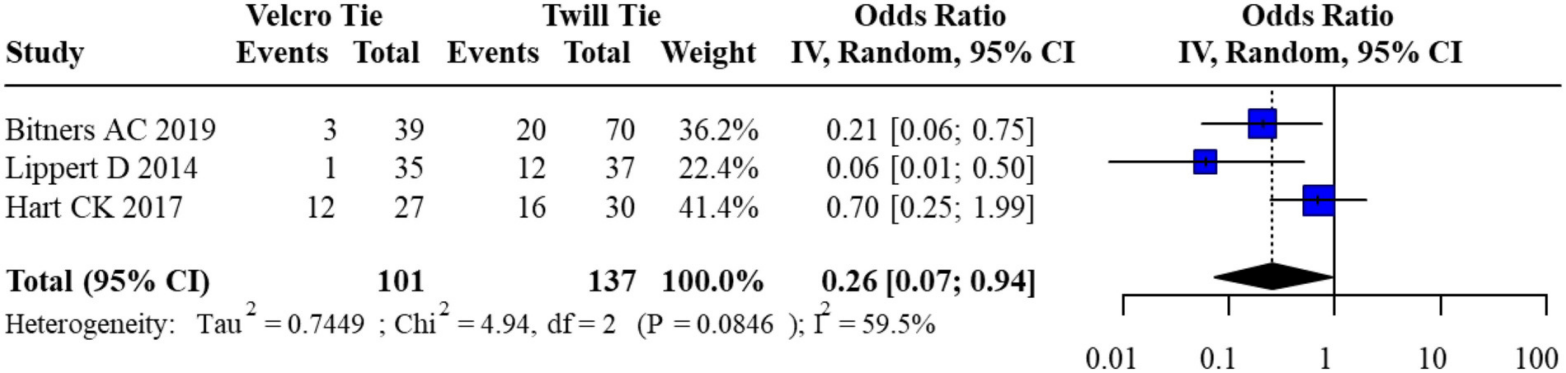
Forest plot comparing pressure injury risk between velcro® ties and standard twill ties.

Notably, the analysis revealed considerable between-study heterogeneity (I² = 59.5%), which may be attributable to variations in study design, participant characteristics, outcome definitions, or institutional nursing protocols across the included investigations. Given that the synthesis included only three studies, the funnel plot (Figure 2) is presented for descriptive purposes only; formal assessment of publication bias or small-study effects could not be reliably performed, and the possibility of publication bias cannot be excluded. Visual representation of the pooled effect estimates is provided in the forest plot (Figure 3).

**Figure 4:**
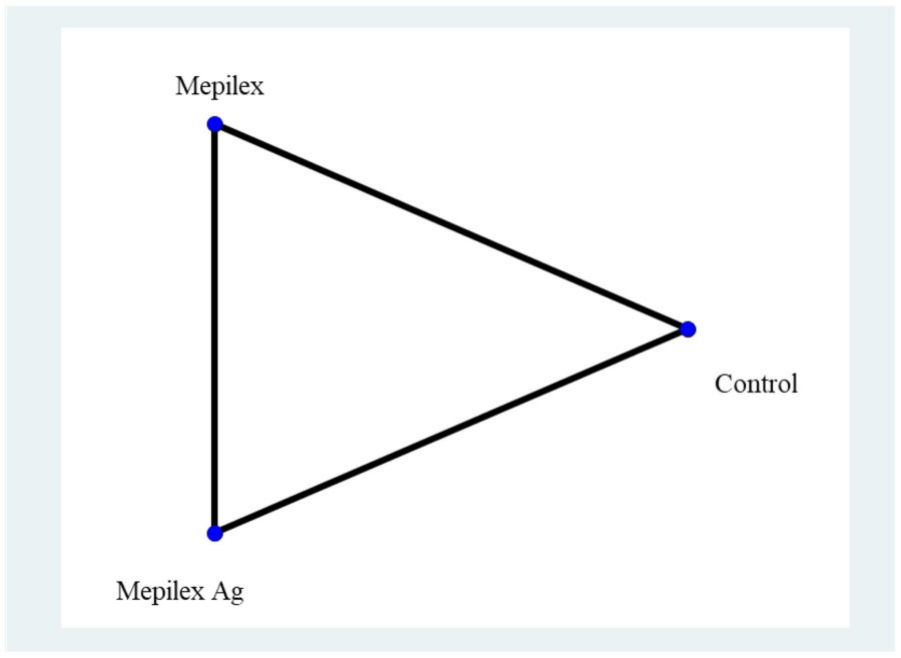
Network geometry plot of comparisons among mepilex®, mepilex® Ag, and standard care for pressure injury incidence.

Subsequently, a network meta-analysis was performed to compare the effects of Mepilex®, Mepilex® Ag, and standard care on pressure injury incidence. This analysis included one study comparing Mepilex® vs. standard care, one comparing Mepilex® Ag vs. standard care, and one providing a direct comparison between Mepilex® and Mepilex® Ag. The network graph (Figure 4) demonstrated good connectivity, with standard care serving as a common comparator linked to both Mepilex® and Mepilex® Ag, and a direct link between the two active dressings provided by Marcet-Gonzalez J (12). Node size reflects the sample size for each intervention, with Mepilex® (*n* = 121) and standard care (*n* = 161) having the largest nodes, followed by Mepilex® Ag (*n* = 93). Edge thickness corresponds to the number of studies providing direct evidence for each comparison; all comparisons were informed by single-study direct evidence.

**Figure 5:**
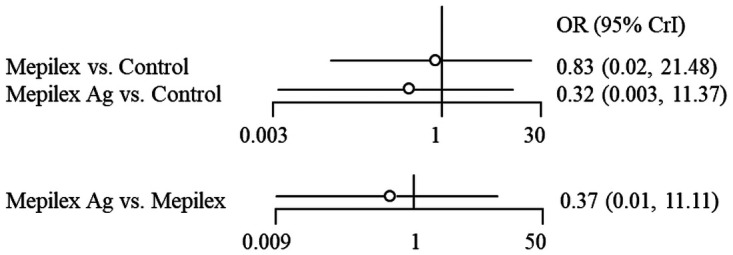
Forest plot of pairwise comparisons for the effects of different interventions on pressure injury incidence.

Compared to standard care, the RR for Mepilex® was 0.83 (95% CrI: 0.02–21.48), and for Mepilex® Ag it was 0.32 (95% CrI: 0.003–11.37). In the head-to-head comparison, the OR for Mepilex® Ag relative to Mepilex® was 0.37 (95% CrI: 0.01–11.11), reported only as OR because this was a network comparison. All CrIs and ORs are consistent with the methods and account for sparse-event data. None of these comparisons reached statistical significance, as indicated by all confidence intervals crossing the null value (OR = 1) and being very wide. This high degree of uncertainty likely stems from the limited number of studies, small sample sizes, and the occurrence of zero events in the Mepilex® Ag group in the Kuo CY (11) study. (Figure 5).

**Figure 6:**
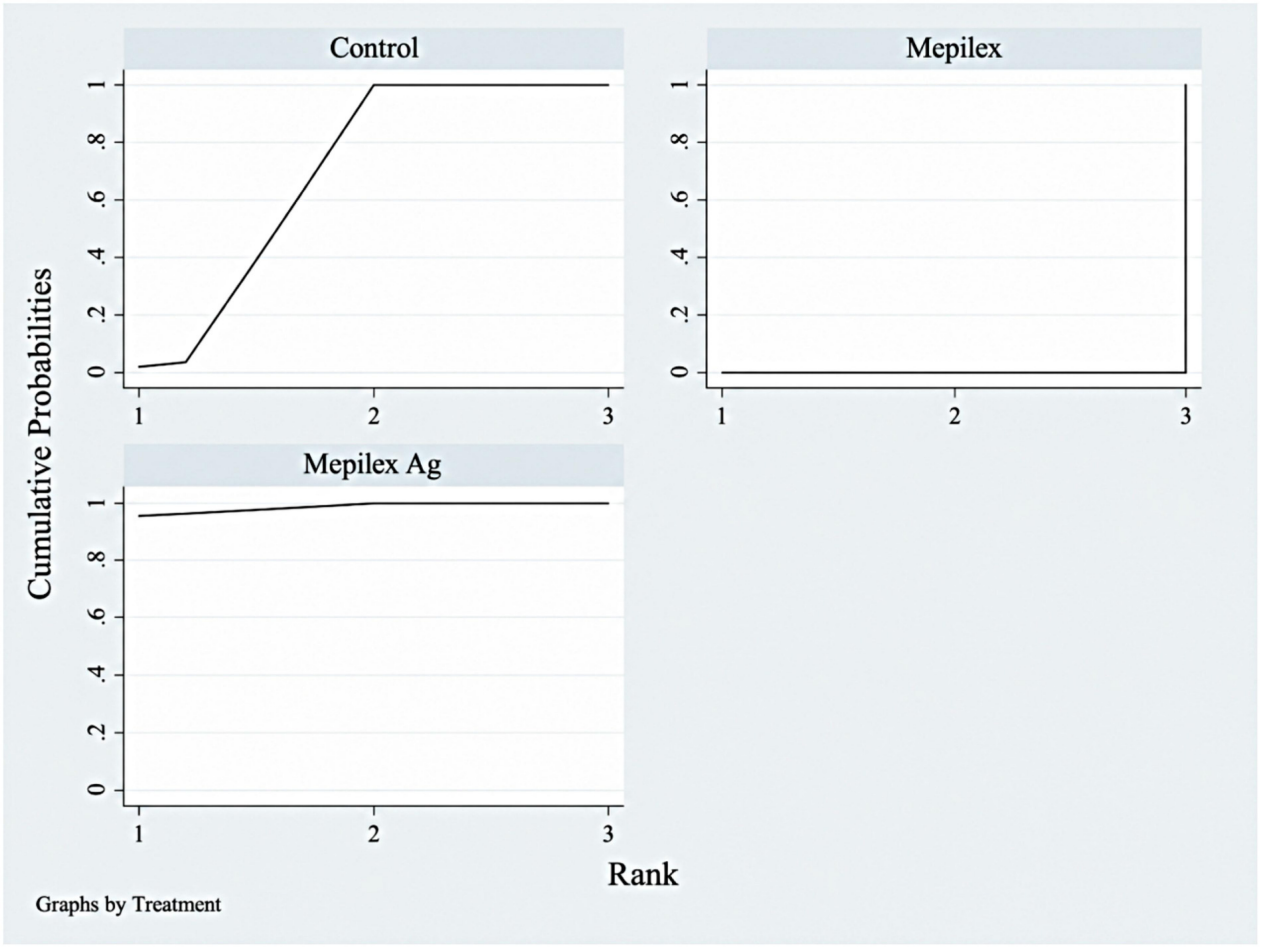
Surface under the cumulative ranking (SUCRA) plot for interventions preventing pressure injuries.

The SUCRA rankings suggested that Mepilex® Ag had a relatively higher probability of being the most effective intervention (SUCRA = 97.6%) compared with standard care (SUCRA = 52.2%) and Mepilex® (SUCRA = 0.1%) (Figure 6). Given the wide credible intervals, sparse network evidence, and lack of statistically significant pairwise comparisons, these SUCRA results should be interpreted with caution and regarded as indicative rather than definitive of comparative effectiveness.

## 4 Discussion

This systematic review aimed to evaluate the effectiveness of various quality improvement strategies for preventing post-tracheostomy MDRPI in children, defined according to NPIAP/EPUAP standards. Through meta-analytic techniques, we compared interventions such as advanced dressings and modified securement devices to provide an evidence base for clinical nursing practice. As pediatric critical care advances and tracheostomy indications shift toward chronic conditions requiring long-term management, effective pressure injury prevention has become a central concern for both patient safety and healthcare quality.

Regarding securement methods, our meta-analysis found that Velcro®-type ties were associated with a lower risk of skin-related adverse events compared to traditional twill ties (RR = 0.26, 95% CrI 0.07–0.94). However, considerable between-study heterogeneity was observed (I² = 59.5%), likely due to variations in study design, participant characteristics, outcome definitions, and institutional nursing protocols. The pooled analysis of three studies, including one RCT, suggested that Velcro® ties may reduce pressure injury risk by approximately 74%. The retrospective study by Bitners et al. (10) supported this finding, reporting reduced skin irritation risk (OR = 0.41) (10). However, the RCT by Hart et al. (9) did not find a statistically significant difference in overall skin complications, although it noted a lower proportion of Velcro®-tie patients requiring early tie change (7.4% vs. 20%) (9). This discrepancy suggests that while observational data favor Velcro® ties, findings from the sole RCT were inconclusive, possibly due to differences in study populations, standardization of care, or outcome definitions.

In the comparison of dressings, the network meta-analysis did not reveal significant differences between Mepilex® and Mepilex® Ag (OR = 0.37, 95% CI 0.01–11.11). This suggests that while silver-containing dressings offer theoretical antimicrobial benefits, their efficacy in specifically preventing pressure injuries may not substantially exceed that of non-silver foam dressings. Given the higher cost of silver-containing variants, clinical decisions should weigh potential benefits against cost-effectiveness. Standard Mepilex®, a non-silver foam dressing, primarily provides protection through its high absorbency and low friction coefficient. A retrospective study by Odom et al. comparing foam dressings alone vs. foam dressings combined with a wound filler found no significant difference in healing time for pressure injuries, though stratified analysis suggested potential benefit for Stage 2 injuries with higher PUSH scores (13). A meta-analysis by Yue et al., synthesizing 10 studies with 1,220 patients, confirmed that moist dressings (including foam) significantly reduced tracheostomy site infection and pressure injury rates compared to traditional gauze, while also decreasing dressing change frequency and shortening healing time (14).

Mepilex® Ag is a dressing composed of a polyurethane foam absorption layer combined with a soft silicone wound contact layer containing silver ions, designed for hydrophilicity, absorbency, and antimicrobial activity. The retrospective study by Kuo et al. (11) was the first to apply it in the pediatric post-tracheostomy population, reporting zero instances of skin breakdown at first tube change in the intervention group (*n* = 41) compared to an 11.8% rate in the standard care group (*n* = 93, *p* = 0.02) (11). This striking result positioned Mepilex® Ag as a significant option for prevention. However, the prospective RCT by Marcet-Gonzalez et al. (12) found that standard Mepilex® was non-inferior to Mepilex® Ag in preventing peristomal complications (non-inferiority *p* = 0.0108), suggesting that the basic foam dressing may provide comparable physical barrier protection, and the additional antimicrobial effect of silver might not translate into a clinical advantage in short-term prophylaxis (12).

Our findings broadly align with the existing literature, though some nuances warrant discussion. The systematic review and meta-analysis by Moser et al. (15), which included 10 studies with 2023 adult and pediatric critically ill patients, reported a substantial reduction in tracheostomy-related pressure injuries from 17.0% to 3.5% (a 79% decrease) following intervention implementation (15). The interventions in that review included foam dressings, hydrocolloid dressings, extended-length tracheostomy tubes, and foam neck braces, with some evidence from RCTs. In contrast, direct comparative studies specifically within the pediatric population remain scarce; our meta-analyses for both Velcro® ties and dressings were limited to three studies each, predominantly observational, highlighting a lag in pediatric-specific research.

Successful quality improvement initiatives in adults offer valuable references for pediatric care. O'Toole et al. (16) implemented a bundle including hydrocolloid dressings, early suture removal, foam dressings, and neutral head positioning, reducing adult tracheostomy-related pressure injuries from 10.93% to 1.29% (*p* = 0.0003) (16). Similarly, the project by Dixon et al. (17) demonstrated an 80% reduction in pressure injuries through standardized suturing techniques, dressing selection, and staff training (17). The interprofessional prevention bundle implemented by Urquhart et al. (18) in critically ill adults resulted in a 50% reduction in the daily rate of tracheostomy-related pressure injuries (18). Its core elements—daily skin assessment, standardized documentation, and risk reporting protocols—closely mirror potential strategies in pediatrics, underscoring the universal value of multidisciplinary collaboration.

It is crucial to acknowledge the distinct anatomical and physiological characteristics of children, especially infants, compared to adults. Pediatric skin features a thinner stratum corneum, more abundant subcutaneous tissue, and a higher pH, rendering it significantly less tolerant of shear forces and moisture (19). Furthermore, children receiving tracheostomies often have complex comorbidities such as neuromuscular diseases or congenital anomalies (20, 21), and may require cannulation for years (22), creating a chronic, cumulative pressure injury risk profile different from that of acutely ill adults. A comparative study by Süslü et al. (23) involving 53 children and 83 adults found that while overall complication rates were similar (22.6% in children vs. 19.3% in adults), the mean time to decannulation was significantly longer in children (317 days vs. 69 days, *p* = 0.040) (23). This emphasizes that the temporal dimension of pressure injury prevention is particularly salient in pediatrics.

Children with head and neck tumors represent a uniquely vulnerable subgroup for post-tracheostomy pressure injuries (24, 25). Rhabdomyosarcoma, the most common soft tissue sarcoma in children, arises in the head and neck region in approximately 35% of cases, making it a frequent diagnosis in school-aged children (26). These children may require tracheostomy due to tumor-related airway obstruction or as part of multimodal treatment involving surgery, radiation, and chemotherapy. Craniofacial radiation can induce microcirculatory damage, delayed collagen remodeling, and localized immunosuppression, severely compromising the tolerance of irradiated skin to pressure, friction, and moisture (27, 28). Concurrent chemotherapy may contribute to malnutrition, metabolic disturbances from nausea and vomiting, and neutropenia, further impairing tissue repair capacity and infection defense. For this high-risk population, clinical teams should implement enhanced preventive strategies beyond routine protocols. These may include more frequent skin assessments during radiation, preferential use of silver-containing foam dressings to combine pressure relief with antimicrobial protection, utilization of Velcro®-type ties to minimize shear, and intensified nutritional support.

The findings of this review hold significant potential for clinical translation. From a pathophysiological standpoint, pressure injuries in children with tracheostomies arise from a combination of pressure, shear, moisture, and microbial colonization. Silver-containing foam dressings offer multifactorial protection by redistributing pressure, absorbing exudate, and providing antimicrobial action, which may be particularly beneficial for children with significant secretions. The advantages of Velcro®-type ties lie in their potential for more even pressure distribution, ease of adjustment, and reusability. However, safety considerations are paramount; while accidental decannulation rates are reported as very low (<2%) in the included studies and literature, careful assessment is warranted in children with neck edema or anatomical abnormalities.

This study has several limitations. First, the small number of included studies, particularly for the network meta-analysis which relied on only 2–3 direct comparisons, resulted in wide confidence intervals and insufficient statistical power to draw definitive conclusions. Second, methodological quality was variable, with only one included RCT (Hart et al.) and the remainder being retrospective cohort or pre-post designs, introducing potential selection bias and confounding. Third, sources of heterogeneity could not be fully explored. Fourth, publication bias is a concern, as positive results may be more readily published, while negative or small studies may be underrepresented; however, the limited number of studies precluded meaningful assessment via funnel plots.

In response to the methodological limitations and knowledge deficits identified in this review, subsequent investigative efforts should be directed toward several critical priorities. Foremost among these is the imperative for well-designed, adequately powered, multisite randomized controlled trials. Such studies are essential to generate the high-certainty evidence required to definitively establish the comparative effectiveness of different preventive strategies and to inform the development of robust clinical practice guidelines for this vulnerable population. These should employ standardized outcome measures (e.g., the EPUAP/NPIAP pressure injury staging system) and include adequate follow-up periods (at least 30 days post-operation or until discharge) to generate robust evidence. Second, technological integration and innovation should be explored. This includes investigating the potential of 3D-printed personalized securement devices, biosensors for real-time monitoring of skin pressure, and artificial intelligence-assisted risk prediction models. Third, rigorous cost-effectiveness analyses comparing different preventive strategies within real-world healthcare settings, considering direct medical costs and long-term quality-adjusted life years (QALYs), are essential to inform health policy and resource allocation.

## 5 Conclusion

This systematic review provides preliminary evidence that silver-containing foam dressings and Velcro®-type securement devices may help prevent tracheostomy-related MDRPI in children. Bundled nursing approaches appear promising. Nevertheless, the certainty of the evidence is moderate according to the GRADE framework, based on the limited number of studies, variable methodological quality, and substantial heterogeneity. Evidence for RCTs was downgraded for imprecision and indirectness, while observational studies were rated low and further downgraded for risk of bias and inconsistency. Clinical decision-makers should interpret these findings with caution, integrating individual patient characteristics, resource availability, and cost-effectiveness considerations. High-quality randomized controlled trials and real-world studies with larger sample sizes are urgently needed to establish robust, evidence-based guidelines for pediatric MDRPI prevention and to improve outcomes for this vulnerable population.

## Data Availability

The original contributions presented in the study are included in the article/Supplementary Material, further inquiries can be directed to the corresponding author/s.
