# Supplementary material for: Quality improvement strategies for preventing tracheostomy-related pressure injuries in children: a systematic review and meta-analysis
Source: Front Pediatr. 2026 Jun 2;14:1829526. doi: 10.3389/fped.2026.1829526 (PMC13269278; doi:10.3389/fped.2026.1829526)
Supplement: Supplementary file 2 [file Supplementaryfile2.docx]

**Search Strategies**

This supplementary material provides the complete database search strategies used in this systematic review and meta-analysis, covering both international and Chinese databases. The search date and the combination of search terms are specified to facilitate reproducibility of the study.
**Search Date:** Up to December 2025

1. PubMed #1 “Tracheotomy”[Mesh] OR “Tracheostomy”[Mesh] OR “airway surgical procedure”[tiab] #2 “Child”[Mesh] OR “Infant”[Mesh] OR “Adolescent”[Mesh] OR “Pediatrics”[Mesh] OR child[tiab] OR infant[tiab] OR baby[tiab] OR babies[tiab] OR newborn[tiab] OR neonat[tiab] OR toddler[tiab] OR preschool[tiab] OR schoolchild[tiab] OR adolescent[tiab] OR teen[tiab] OR youth[tiab] OR pediatric[tiab] OR paediatric[tiab] #3 “Pressure Ulcer”[Mesh] OR “Pressure ulcer”[tiab] OR pressure injur[tiab] OR bed sore[tiab] OR decubitus[tiab] OR skin breakdown[tiab] OR peristomal breakdown[tiab] OR peristomal complication[tiab] OR stoma complication[tiab] OR device-related pressure injur*[tiab] OR wound[tiab] OR skin damage[tiab] OR skin irritation[tiab] OR securement[tiab] OR foam dressing[tiab] OR silicone dressing[tiab]

#4 #1 AND #2 AND #3

1. Embase (via Ovid) (‘tracheotomy’/exp OR ‘tracheostomy’/exp OR ‘airway surgical procedure’:ti,ab) AND (‘child’/exp OR ‘infant’/exp OR ‘adolescent’/exp OR ‘pediatrics’:ti,ab OR child:ti,ab OR infant:ti,ab …) AND (‘pressure ulcer’/exp OR ‘pressure ulcer’:ti,ab OR ’pressure injur*‘:ti,ab OR ’bed sore’:ti,ab …)
2. Cochrane Library (CENTRAL) ([MeSH descriptor: “Tracheostomy”] OR tracheostomy OR tracheotomy) AND (child* OR infant* OR pediatric* OR adolescent*) AND (pressure ulcer* OR pressure injur* OR bed sore* OR skin breakdown* OR wound*)
3. Web of Science Core Collection TS=(tracheostomy OR tracheotomy OR “airway surgical procedure”) AND TS=(child* OR infant* OR pediatric* OR adolescent*) AND TS=(“pressure ulcer” OR “pressure injury” OR “bed sore” OR “skin breakdown” OR wound*)
4. CINAHL ((MH “Tracheostomy”) OR tracheostomy OR tracheotomy) AND ((MH “Pediatrics”) OR child* OR infant* OR adolescent*) AND ((MH “Pressure Ulcer”) OR “pressure ulcer” OR “pressure injur” OR ”bed sore” OR “skin breakdown” OR wound*)
5. CNKI (中国知网) 主题: (气管切开 OR 气管造口) AND 主题: (儿童 OR 小儿 OR 新生儿) AND 主题: (压疮 OR 压力性损伤 OR 皮肤破损 OR 皮肤损伤)
6. Wanfang Data (万方数据) 主题检索: (气管切开 OR 气管造口) AND (儿童 OR 小儿 OR 新生儿) AND (压疮 OR 压力性损伤 OR 皮肤破损 OR 皮肤损伤)
7. VIP (维普) 主题检索: (气管切开 OR 气管造口) AND (儿童 OR 小儿 OR 新生儿) AND (压疮 OR 压力性损伤 OR 皮肤破损 OR 皮肤损伤)

**Notes:**

- The search strategies combined both subject headings and free-text terms.
- The search date was up to December 2025.
- No language restrictions were applied in any of the database searches.
